# The Ketogenic Diet in Children with Epilepsy: A Focus on Parental Stress and Family Compliance

**DOI:** 10.3390/nu15041058

**Published:** 2023-02-20

**Authors:** Francesca Felicia Operto, Angelo Labate, Salvatore Aiello, Cristina Perillo, Valeria de Simone, Rosetta Rinaldi, Giangennaro Coppola, Grazia Maria Giovanna Pastorino

**Affiliations:** 1 Child and Adolescent Neuropsychiatry Unit, Department of Medicine, Surgery, and Odontoiatry, University of Salerno, 84084 Salerno, Italy; 2 Neurophysiopatology and Movement Disorders Clinic, University of Messina, 98122 Messina, Italy

**Keywords:** parental stress, ketogenic diet, children, epilepsy

## Abstract

(1) Background: The aim of our study was to evaluate parental stress after 6 and 12 months of a ketogenic diet, considering demographic and clinical variables (epilepsy type, epilepsy duration, seizure number, antiseizure medications, comorbidities, efficacy, and adverse events). (2) Methods: We consecutively enrolled 36 children aged between 3 and 10 years who had been diagnosed with various types of drug-resistant epilepsy and who were in therapy with a ketogenic diet for better seizure control. A standardized neuropsychological questionnaire (Parenting Stress Index–PSI) was administered to the parents evaluating parental stress at baseline (T0), after 6 (T1) months, and after 12 months (T2). (3) Results: After 6 and 12 months of dietary treatment, Parental Distress and Total Stress mean scores were statistically significantly increased. Post hoc analysis showed no significant changes in the scores between T0 and T1, although there was a significant increase between T1 and T2. We did not find statistically significant relationships between parental stress and the other variables considered. (4) Conclusions: The ketogenic diet can be challenging for parents and can affect the perception of parental stress, especially in the long term. Parents may feel inadequate in their role; therefore, they should be helped and encouraged through additional supports in order to maximize the adherence to diet therapy.

## 1. Introduction

The ketogenic diet (KD) is a high-fat and low-carbohydrate and -protein nutritional regimen which is used as an alternative treatment for both focal and generalized drug-resistant epilepsies. [1]. Currently, despite the introduction of new generation antiseizure medications, about 25–30% of patients with epilepsy are drug resistant (Kwan et al., 2009); therefore, the interest in the KD continues to remain high. Several recent studies confirmed the positive effects of the KD in refractory epilepsies both in adult and in pediatric populations [2]. 

The KD is a strictly and personally calculated diet, regularly monitored in order to evaluate ketosis and metabolic–nutritional status [3]. The classic KD is high in lipids (approximately 90% of daily caloric intake, predominantly in the form of long-chain fatty acids) and low in carbohydrates and proteins (approximately 1 g/kg body weight, to ensure adequate growth in pediatric patients). The KD can require an integration with supplements of multivitamins and minerals. Generally, the ideal ratio between the amount of lipids and carbohydrates plus protein is 4:1 (which indicates that each gram of carbohydrates corresponds to 4 g of fats); however, a 3:1 ratio may be used as needed by the patient [1]. A key aspect of the KD includes partial caloric restriction (10 to 25% of total daily caloric intake), which is necessary to maintain the state of ketosis and, thus, achieve the effects. In the past years, the initiation of the diet was preceded by a 24–48 h fasting period, which required hospital monitoring for the possibility of hypoglycemia and dehydration; over the past several years, the fasting phase has been eliminated with a gradual weekly increase in the ratio of nutrients from 1:1 to 4:1 [1]. More flexible KD schemes have been developed over the years to achieve better patient compliance and reduce adverse effects [1]. In the last decades, in addition to the classic KD, new variants have emerged, such as the modified Atkins diet (MAD) and the low index diet glycemic (LGIT), which allow a higher protein content and do not require calorie or liquid restriction [1,2]. The dietary scheme is individualized on the basis of patient characteristics such as age, weight, family characteristics, type of epilepsy. And comorbidities [1]. 

Current knowledge of the actual mechanisms through which the KD acts on the reduction in epileptic discharges is still incomplete. It seems increasingly plausible that, at the basis of the therapeutic success of the KD, there is a combination of multiple factors rather than a single mechanism of action and that all contribute, directly or indirectly, to regulating neuronal metabolism and cortical hyperexcitability [3]. Among the factors best described are those related to a decrease in glucose levels and an increase in ketone bodies in blood and cerebrospinal fluid. Recent studies have highlighted increasingly more important roles of functionality mitochondrial, neuroinflammation, and modification of the intestinal microbiota compared to other factors.

Traditionally, the KD has been considered the gold standard for the treatment of metabolic diseases such as GLUT-1 and pyruvate dehydrogenase deficiency syndrome [2]. To date, this treatment is reported to be more effective than other treatments in different clinical conditions such as drug-resistant epilepsy, infantile spasms, Lennox–Gastaut syndrome, Dravet syndrome, myoclonic–astatic epilepsy, and tuberous sclerosis complex [4,5,6,7]. In addition, literature studies reported beneficial effects of the KD in patients with symptomatic epilepsies waiting for surgery or for a pre-surgical evaluation [8]. Acute adverse effects of the KD include dehydration, hypoglycemia, lethargy, metabolic acidosis, and gastrointestinal symptoms. However, the most frequently occurring side effects are weight loss, increased low-density lipoprotein, and increased cholesterol. Other side effects are gastrointestinal symptoms, including constipation, diarrhea, vomiting, and abdominal pain. The development of nephrolithiasis is possible, requiring ultrasound monitoring. MAD has demonstrated a better tolerability profile with some typical side effects such as gastrointestinal disturbances, dyslipidemia, and weight loss. Constipation and vomiting are the most common side effects reported in LGIT patients [9]. Before the KD is started, a preliminary visit is strongly recommended, the objectives of which are the clinical overview of the neurological disorder and the exclusion of any contraindications to the KD. Before starting treatment, it is also crucial to carry out adequate parental counseling, as a key component in the compliance and long-term maintenance of the KD is the information that the family receives before starting the diet. Adequate communication to parents/caregivers is required about expectations in terms of clinical improvement or a reduction in antiseizure drugs, the minimum time period necessary to realize the effects, and potential management difficulties in the short and long term [10,11].

In recent years, due to the growing use of the KD in various clinical settings, food companies have begun to develop numerous ketogenic products. This aspect has contributed to improving the quality of life of patients and their families, expanding the range of products to choose from and increasing food variability. Currently, it is possible to have access to alternative foods that are very similar to traditional ones, and which, especially in the pediatric field, enable young patients to feel less different from their peers, increasing compliance. Furthermore, new basic ingredients are available for preparing recipes that would otherwise be difficult to follow and which are very useful, especially for patients undergoing the KD for prolonged periods [12,13,14].

Despite the progress of recent years, nutritional therapies for pediatric patients and patients with disabilities require intense parent/caregiver commitment [15]. Parents are responsible for managing the diet; therefore, parental compliance is the main factor influencing adherence to diet therapy. 

Undertaking the KD affects eating and social habits and can significantly change the quality of life of the entire family unit. Parents of children following the KD may experience some difficulties related to various factors such as the child’s poor compliance with the diet, social repercussions, a lack of time, or a lack of clinical support [15]. These factors can affect parental stress and lead to a discontinuation of therapy [15]. On the other hand, the effectiveness of therapy and the reduction in seizure frequency can reduce parental stress levels [16].

The aim of our study was to evaluate the change in parental stress levels after 6 and 12 months of the KD, also analyzing some demographic and clinical variables (type of epilepsy, duration of epilepsy, number of seizures, number of antiseizure medications, comorbidities, efficacy of the KD, adverse events of the KD).

## 2. Materials and Methods

### 2.1. Study Design

This is a longitudinal monocentric observational study that evaluates parental stress levels in parents of children and adolescents with epilepsy in therapy with the ketogenic diet.

### 2.2. Participants

We consecutively enrolled children aged between 3 and 10 who had been diagnosed with various types of drug-resistant epilepsy and who were in therapy with a ketogenic diet at the Child and Adolescents Neuropsychiatry Unit of the University of Salerno from June 2019 to June 2021.

The patients were evaluated by two expert clinicians, who diagnosed epilepsy according to the most recent classification of the International League Against Epilepsy (ILAE 2017) [17]. All patients underwent a clinical evaluation to assess seizure characteristics, video-EEG (v-EEG), and brain MRI if it was needed. 

Exclusion criteria included poor parental compliance to the standardized neuropsychological assessment. We evaluated parental stress at baseline (T0), after 6 months (T1), and after 12 months (T2) through the administration of a standardized neuropsychological questionnaire. Parents were also provided with a non-standard questionnaire in which to report the major difficulties encountered in carrying out the ketogenic diet.

Compliance with diet therapy was assessed by measuring ketonemia and glucose blood levels. We considered also the following clinical features: age at onset of epilepsy, epilepsy duration, seizure numbers, antiseizure medication type and numbers, response to the ketogenic diet.

The aims and methods of the study were explained in detail to the patients and their parents. Written informed consent was obtained from all parents. The protocol was approved by the local ethics committee, “Campania Sud”, according to the rules of good clinical practice, in line with the Declaration of Helsinki.

### 2.3. Parenting Stress Index—Short Form

The Parenting Stress Index—Short Form (PSI) [18] is a standardized questionnaire addressed to parents of children and adolescents that measures parental stress levels. This form of PSI is composed of 36 sentences, which parents can evaluate on the basis of the following scale: “5 = strongly agree ” to “1 = strongly disagree”.

The PSI/SF provides three subscales, each of which is composed of 12 items:

- Parental Distress (PD), which represents the stress related to the parenting role. This subscale measures the level of stress that the parent is experiencing in his role, which is understood to be derived from personal factors directly related to that role (e.g., responsibility in managing the different aspects related to the child’s pathology; personal life limitations related to child management).
- Dysfunctional Parent–Child Interaction Scale (P-CDI), which assesses the level of stress related to parent–child interaction. This subscale measures the level of stress that occurs when the parent perceives the child as not responding to their expectations, and interactions with the child do not reinforce them as a parent (e.g., my child fails to do as well as I expected; I have the feeling that my efforts are not much appreciated).
- Difficult Child Scale (DC) which assesses the stress related to characteristics of the child. This subscale measures the level of stress related to the child’s peculiar characteristics which render them difficult to manage and which often originate in their temperament, including defiant and disobedient behaviors (e.g., crying, irritability, problematic behaviors, problem of sleep, problem of feeding).

Finally, from these three subscales, it is possible to provide a main scale which measures the levels of Total Stress (TS). 

In PSI/SF, the raw score is converted to t-scores; a t-score ≥85 highlights clinically significant parental stress. 

### 2.4. Statistical Analysis 

We expressed the neuropsychological scores as mean ± standard deviation (SD). We preliminarily performed the Kolmogorov–Smirnov normality test to verify the data distribution. Due to the presence of data not normally distributed, we had to use non-parametric tests for our analysis. The comparison of the mean scores at different times was performed using the Friedman analysis of variance test for the paired sample. Post hoc analysis was performed through the Wilcoxon signed ranked test. The Spearman correlation analysis was employed to evaluate the relationship between different variables and parental stress. All data were analyzed using Statistical Package for Social Science software (Version 25.0, SPSS Inc., Armonk, NY, USA) (IBM Corp., Armonk, NY, USA, Released, 2017); *p*-values less than or equal to 0.05 are considered statistically significant.

## 3. Results

### 3.1. Participants 

Thirty-six children aged between 3 and 10 years (20 male, 16 female) with various forms of drug-resistant epilepsy were enrolled in our study. All clinical and demographic characteristics of the patients are summarized in Table 1 and Table 2. All the patients started a ketogenic diet for incomplete seizure control with two or three antiseizure medications (mean 2.64 ± 0.49). All the patients followed a 4:1 ketogenic diet (Table 2) for the entire period of the study. The mean age at diet initiation was 6.84 ± 2.17 years (3–10.5 years); the mean age of seizure onset was 2.42 ± 0.92 years (range 1–3.5 years); and the mean seizure frequency at baseline was 8.73 ± 8.45 per day (range 0.3–24 per day). The majority of our patients (17/36, 47%) were diagnosed with generalized epileptic encephalopathy due to hypoxic–ischemic neonatal injury (11/36, 31%) or unknown (3/36, 8%). Four patients (11%) were diagnosed with symptomatic focal epilepsy with secondary generalization, and in all of them, we documented neuronal migration disorders on MRI examination. Four patients (11%) were diagnosed with Lennox–Gastaut syndrome, and two patients were diagnosed with West syndrome (6%). Genetic diagnosis was performed for five patients (14%) with tuberous sclerosis complex, for three patients (8%) with Dravet syndrome, and for one patient (3%) with Angelman syndrome. All patients had psychomotor developmental delay/intellectual disability ranging from mild/moderate to severe, as measured by standardized neuropsychological assessment. 

After six months of follow-up of the ketogenic diet, 5/36 (14%) patients discontinued therapy, 4 of them due to poor seizure control and 1 of them due to poor parental compliance. After 12 months of therapy, 3/36 (8%) patients discontinued the ketogenic diet due to poor seizure control.

Most patients (28/36, 78%) remained on the ketogenic diet, and after 12 months, they showed a seizure reduction >50% (23/36, 64%) or were seizure-free (5/36, 14%). No increase in seizure frequency was observed in any patient. 

Adverse events due to the ketogenic diet occurred in 23/36 (64%) of patients. Often, different adverse events could occur simultaneously in the same subject, but all the adverse events were temporary and mild, and they did not lead to discontinuation of the diet. The adverse events that occurred most frequently in the first month of the ketogenic diet were constipation, drowsiness, lack of appetite, nausea/vomiting, and diarrhea. The late adverse events, which occurred after 3–6 months on the ketogenic diet, were weight loss, vomiting, or gastroesophageal reflux. Adverse events and their frequency in our sample are summarized in Table 3.

### 3.2. Comparison of Parental Stress at Baseline after 6 and 12 Months on the Ketogenic Diet

The evaluation of parental stress through the standardized neuropsychological questionnaire PSI showed that parental stress was elevated at baseline (T0) in all the analyzed scales (Parental Distress, Dysfunctional Parent–Child Interaction, Difficult Child, and Total Stress), with average scores in the pathological range if compared to the standard reference values.

After 6 (T1) and 12 (T2) months of dietary treatment, Parental Distress and Total Stress mean scores were statistically significantly increased. Post hoc analysis showed that there were no significant changes in the scores between T0 and T1, whereas there was a significant increase between T1 and T2. All the mean scores of the PSI and the results of the statistical comparison are reported in Table 4 and Table 5. Spearman’s correlation analysis did not show statistically significant correlations between levels of parental stress and other variables such as age, age of onset of seizures, duration of epilepsy, frequency of seizures, number of antiseizure medications, and seizure reduction (*p* > 0.05). The main reasons for parental difficulties in managing the ketogenic diet are reported in Table 6.

## 4. Discussion

The ketogenic diet can represent an important commitment for parents/caregivers of pediatric patients from an organizational, social, economic, and also psychological point of view [15]. The change of eating habits, the lack of flexibility, and the strictly planned and obligatory meals can be stress factors for the parents and for the whole family.

The objective of our observational study was to evaluate the changes of parental stress after 6 and 12 months of the ketogenic diet through a standardized neuropsychological assessment.

The parental stress analysis showed that, in our sample, parental stress levels were already high at baseline in all the scales of the Parenting Stress Index (Parental Distress, Parent–Child Dysfunctional Interaction, Difficult Child, and Total Stress), highlighting that there were different stress factors related to the parental role, to the parent–child relationship, and finally, to the individual characteristics of the child (Table 4). This data could be explained considering that all our patients suffered from severe clinical conditions, with recurrent seizures not completely controlled by drug therapy and psychomotor developmental delay/intellectual disability [19,20,21]. Our data confirm previous literature data, which showed that parents of children with chronic medical conditions are at risk of experiencing high stress levels [22]. In particular, parents of children with epilepsy experience high levels of concern about the future recurrence of seizures, the difficulty in managing the antiseizure medication, the adverse events of drug therapy, the potential comorbidities, and the social stigma [23,24,25,26]. 

Analysis of parental stress over time showed an increase in parental stress after both 6 and 12 months of the ketogenic diet, although only the latter variation was statistically significant. This finding suggests that parental stress increases as months go by, and the commitment to properly maintain the ketogenic diet becomes more challenging over time (Table 4 and Table 5). 

Analyzing the individual PSI subscales, we found that Parental Distress and Total Stress scores were statistically significant increased, whereas the increase in Difficult Child and Parent–Child Dysfunctional Interaction scores was not significant. This finding suggests that parental stress levels increased mainly due to difficulties for the parents in coping with their parenting role, rather than the child-related characteristics (Table 4 and Table 5). As reported by the parents (Table 6), in our sample, the main factors that negatively affect parental stress levels were the difficulties in management (which included finding and preparing food, monitoring blood glucose and ketonemia, periodic clinical checks); the lack of time (preparation and administration of meals); the child’s compliance (poor appetite, refusal of food); the adverse events; the economic factor; the social/outing restrictions (difficulty in attending public places, decrease in leisure opportunities); and finally, the need for greater clinical support. 

Correlation analysis showed that the clinical and demographic factors that we have considered did not appear to significantly affect parental stress levels. These results suggest that parental stress increases regardless of the type of epilepsy, the duration of epilepsy, the number of seizures, the number of antiseizure medications, comorbidities, efficacy, and adverse events of the ketogenic diet. Furthermore, surprisingly, parental stress at 6 and 12 months did not correlate significantly with a reduction in seizure frequency. In fact, although in our sample, most of the patients obtained a >50% seizure reduction, and five patients were seizure-free, we observed an increase in parental stress. These apparently conflicting results could be explained by the fact that the increase in parental stress mainly concerns the “Parental Distress” subscale, which investigates the stress derived from parental factors instead of the characteristics of the child. From this point of view, we can hypothesize that the parents feel a growing responsibility in the management of their children’s pathology. Even if the ketogenic diet in our sample brought good results in terms of seizure reduction, it could have generated a psychological load on the parents due to the difficulties in maintaining clinical results over time. 

It should also be considered that the patients in our sample followed a 4:1 ketogenic diet for the entire duration of the study in order to maintain good efficacy on seizure control. Therefore, because a 4:1 ketogenic diet requires strict food restrictions, this aspect could have negatively influenced family management and parental stress.

These results are in agreement with those from some previous studies. In particular, a retrospective study by Tong et al. (2022) [27] collected and analyzed clinical data of children with drug-resistant epilepsy on a ketogenic diet (retention rate, efficacy, side effects of the KD, and the reasons for discontinuation of the KD) at the baseline and during follow-up at 1, 3, 6, 12, 18, and 24 months. The authors concluded that despite increasing availability and good efficacy, long-term adherence to the KD was difficult. Compliance issues appeared to be prominent. In fact, less than half of the patients adhered to the KD for one year, and about 1/5 of the patients adhered for two years. They also suggested that enhancing food taste and patient support can help improve the retention rate.

Our study is also in agreement with previous studies in the literature; in particular, a survey by Sarlo and colleagues (2021) [15] on a sample of 192 participants showed that 76% of parents/caregivers classified dietary therapy as “very challenging”, and 99% reported at least one difficulty in the management of dietary therapies. The authors concluded that caregivers reported substantial difficulties for themselves and their children while using the dietary treatment for epilepsy; however, they felt supported and were happy with the therapy and the improved quality of life for their children, suggesting that the benefits outweigh the difficulties.

The results of our study disagree with the findings of the study by Pulsifer et al. (2001) [16], in which parental stress levels after 1 year of follow-up in the parents of children with drug-resistant epilepsy (n = 34) were substantially unchanged, probably due to good success in seizure reduction. This disagreement could be due to the socio-economic changes that have occurred over the last 20 years and to the different perception of the quality of life of the families [28,29]. Furthermore, in this regard, we should also consider that our study was conducted during the COVID-19 pandemic, and this may have been an additional factor that impacted parental stress [30,31,32]. Some recent research showed that the COVID-19 pandemic can be a source of parental stress in parents of children and adolescents with various neuropsychiatric conditions, including epilepsy [33,34]. On the other hand, an observational study by Armeno and colleagues (2022) [35] did not highlight additional difficulties in managing the KD during the COVID-19 pandemic and supported the feasibility and safety of initiating and managing the KD by telemedicine. The authors explored the feasibility, effectiveness, and safety of the KD in children with drug-resistant epilepsy compared to a group of children followed by telemedicine (n = 18) and a group of children followed in an outpatient modality (n = 19). Dietary compliance, ketosis, retention rate, adverse effects, and clinical outcome were evaluated at 1, 3, and 6 months on the diet, and no statistical differences between the two groups regarding the efficacy and safety of the diet were found.

The main limitations of this study are the small sample size and the lack of a control group. Furthermore, the PSI-SF does not allow us to differentiate parental stress due to the ketogenic diet and parental stress due to epilepsy itself, so it would be useful to further investigate this aspect thought qualitative questionnaires/interviews for the parents in future research. In addition, in future studies, other variables (such as the neuropsychological, emotional, and behavioral characteristics of children) could be analyzed through a standardized assessment. We also aim to perform a new study following a second group of parents of children on the ketogenic diet when the COVID-19 factor will be no longer relevant.

In conclusion, the ketogenic diet can be challenging for parents and affect the perception of parental stress, especially in the long term. Parents may feel inadequate in their role; therefore, they should always be helped and encouraged through additional supports. The clinician can increase the support to parents/caregivers by creating support groups, providing educational material on paper or the web (the literature for dietary therapies for the public), suggesting organizational strategies, ensuring the assistance of the dietician, using mobile phone applications that facilitate management, and also monitoring parental stress levels in order to maximize the success and the adherence to diet therapy in children with epilepsy.

## Figures and Tables

**Table 1:** Demographic and clinical characteristics of children treated with ketogenic diet.

Sex (Male:Female)	20:16
Mean Age at the Diet Onset	6.84 ± 2.17
Diagnosis	
Generalized epileptic encephalopathies	17 (47%)
Focal symptomatic epilepsy	4 (11%)
West syndrome	2 (6%)
Dravet syndrome	3 (8%)
Lennox–Gastaut syndrome	4 (11%)
Angelman syndrome	1 (3%)
Tuberous sclerosis complex	5 (14%)
Psychomotor Delay/Intellectual Disability	
Mild to moderate	15 (42%)
Severe	16 (44%)
Profound	5 (14%)
Cerebral Palsy	
Tetraparesis–diplegia	16 (44%)
Hemiparesis	1 (3%)
Hypotonic syndrome	2 (6%)
MRI Findings	
Tubers	5 (14%)
Neuronal migration disorder	4 (11%)
Hypoxic–ischemic damage	16 (44%)
Aspecific or normal	7 (19%)

**Table 2:** Characteristics of the 36 patients and clinical outcome from the ketogenic diet.

Patient Number	Sex	Age *	Seizure Onset *	Epilepsy Duration *	Diagnosis	Aetiology	Seizure Frequency	ASMs	Type of Ketogenic Diet	Response to the KD (Seizure Reduction % after 12 Months)
1	M	10.5	1.5	9	GE	symptomatic	D	VPA, LTG	4:1	>50
2	F	8.5	2	6.5	GE	symptomatic	D	PB, VPA, LTG	4:1	>50
3	M	5.5	2	3.5	GE	unknown	MD	VPA, LTG, ETS	4:1	<50
4	F	4	3	1	TSC	genetic	MD	CBZ, LTG, CLB	4:1	100
5	F	8.8	3.5	5.3	TSC	genetic	W	CBZ, VGB, CLB	4:1	>50
6	M	8	2.5	5.5	GE	symptomatic	D	VPA, LTG	4:1	>50
7	M	2.5	0.5	2	West	unknown	D	VPA, VGB	4:1	<50
8	F	6.5	3	3.5	LGS	symptomatic	MD	CBZ, TPM, CLO	4:1	<50
9	M	7.7	1.5	6.2	GE	symptomatic	MD	VPA, LTG, PB	4:1	>50
1F	F	4	2	2	LGS	symptomatic	MD	FLB, VPA, LTG	4:1	>50
11	F	9.5	1.5	8	GE	symptomatic	D	VPA, LTG	4:1	>50
12	M	9	2.5	6.5	GE	unknown	D	PB, VPA, LTG	4:1	>50
13	M	5.5	2	3.5	TSC	genetic	D	CBZ, LTG, CLB	4:1	100
14	F	6.5	3.5	3	TSC	genetic	W	VPA, VGB, CLO	4:1	>50
15	M	7	2.5	4.5	FSG	symptomatic	D	VPA, LTG, CLB	4:1	>50
16	F	9.5	1.5	8	GE	symptomatic	D	LEV, PB	4:1	<50
17	F	7.5	2	5.5	GE	symptomatic	MD	VPA, LTG	4:1	>50
18	F	6	0.5	5.5	GE	symptomatic	MD	LEV, VPA	4:1	100
19	M	6.6	1.5	5.1	LGS	symptomatic	MD	VPA, LTG, CLB	4:1	>50
2F	F	3	0.5	2.5	LGS	unknown	MD	VPA, CLB	4:1	>50
21	M	7.5	1.5	6	GE	symptomatic	W	VPA, LTG, ETS	4:1	<50
22	F	9.5	2.5	7	GE	symptomatic	D	LEV, PB	4:1	>50
23	M	7	1.5	5.5	Angelman	genetic	D	VPA, ETS, CLZ	4:1	>50
24	F	8.5	2	6.5	FSG	symptomatic	W	LCS, LEV, CLB	4:1	>50
25	M	6	1	5	Dravet	genetic	MD	VPA, STR, CLB	4:1	>50
26	F	6.5	1.5	5	GE	symptomatic	MD	VPA, LTG	4:1	<50
27	M	9.5	1	8.5	Dravet	genetic	MD	STP, LTG, CLB	4:1	<50
28	M	6	0.5	5.5	Dravet	genetic	MD	VPA, STP, CLB	4:1	>50
29	M	10	2	8	GE	unknown	MD	VPA, PB, LTG	4:1	>50
3F	M	5.5	1.5	4	FSG	symptomatic	MD	LCS, LEV, CLO	4:1	100
31	M	4	2.5	1.5	TSC	genetic	W	CBZ, VGB, CLB	4:1	>50
32	M	10	3.5	6.5	FSG	symptomatic	W	LCS, LEV, CLB	4:1	>50
33	F	4.5	2.5	2	GE	symptomatic	D	PB, TPM, CLB	4:1	>50
34	M	6.5	3.5	3	GE	symptomatic	D	VPA, VGB, CLB	4:1	<50
35	F	6	2,5	3.5	GE	unknown	D	VPA, LTG, CLB	4:1	100
36	M	3	0.5	2.5	West	symptomatic	D	VGB, VPA	4:1	>50
mean ± SD		6.84 ± 2.17	1.93 ± 0.90	4.91 ± 2.12						

* in years. ASM—antiseizure medication; KD—ketogenic diet; M—male; F—female; GE—generalized epileptic encephalopathy; TSC—tuberous sclerosis complex; LSG—Lennox–Gastaut syndrome; FSG—focal secondary generalized epilepsy; D—daily; MD—multi-daily; W—weekly; VPA—valproic acid; LTG—lamotrigine; PB—phenobarbital; ETS—ethosuximide; CBZ—carbamazepine; VGB—vigabatrin; CLB—clobazam; CLO—clonazepam; FLB—felbamate; LEV—Levetiracetam; STP—stiripentol; SD—standard deviation.

**Table 3:** Early and late adverse events on ketogenic diet as reported by parent/caregiver.

Total Patients with at Least One Early or Late Adverse Event on Ketogenic Diet = 23 (64%)	
Early	Late
Constipation = 17 (47%)	Weight Loss = 7 (19%)
Drowsiness/lethargy = 10 (28%)	Vomiting = 3 (8%)
Lack of appetite/food refusal = 9 (25%)	Gastroesophageal reflux = 1 (3%)
Nausea/vomiting = 9 (25%)	
Diarrhea = 7 (19%)	
Abdominal pain = 4 (11%)	
Irritability = 3 (8%)	
Dehydration = 2 (6%)	
Adverse events are often combined in the same patient.	

**Table 4:** Statistical comparison of parental stress at baseline and after 6 and 12 months.

Parenting Stress Index (PSI)				
	Baseline (Mean ± SD) n = 36	6 Months (Mean ± SD) n = 31	12 Months (Mean ± SD) n = 28	Statistical
Parental Distress (PD)	90.41 ± 7.31	92.58 ± 5.30	94.64 ± 5.26	**F = 7.373 *p* = 0.025**
Parent–Child Dysfunctional Interaction (P-CDI)	89.58 ± 9.05	92.58 ± 5.61	93.04 ± 5.50	F = 1.000 *p* = 0.607
Difficult Child (DC)	89.86 ± 8.90	90.97 ± 9.35	90.71 ± 7.66	F = 0.269 *p* = 0.269
Total Stress (TS)	90.83 ± 7.32	93.39 ± 5.23	95.54 ± 4.58	**F = 6.735 *p* = 0.034**

*p*-values that are statistically significant are in bold.

**Table 5:** Post hoc analysis of Parental Distress and Total Stress scales.

	T0 vs. T1	T1 vs. T2	T0 vs. T2
Parental Distress (PD)	W = 1.084 *p* = 0.278	**W = 2.588 *p* = 0.010**	**W = 2.316 *p* = 0.021**
Total Stress (TS)	W = 1.071 *p* = 0.091	**W = 2.652 *p* = 0.008**	**W = 2.735 *p* = 0.006**

*p*-values that are statistically significant are in bold.

**Table 6:** Caregiver difficulties in the management of the ketogenic diet.

difficulties in management	26 (72%)
lack of time	23 (64%)
child compliance	20 (55%)
adverse events	15 (42%)
cost	8 (22%)
social/outing restrictions	7 (19%)
none	6 (17%)
lack of clinical support	3 (8%)

## Data Availability

The data presented in this study are available on request from the corresponding author.
